# *Giardia lamblia* Transcriptome Analysis Using TSS-Seq and RNA-Seq

**DOI:** 10.1371/journal.pone.0076184

**Published:** 2013-10-07

**Authors:** Mohammed E. M. Tolba, Seiki Kobayashi, Mihoko Imada, Yutaka Suzuki, Sumio Sugano

**Affiliations:** 1 Department of Medical Genomics, Graduate School of Frontier Sciences, The University of Tokyo, Tokyo, Japan; 2 Department of Parasitology, Faculty of Medicine, Assiut University, Assiut, Egypt; 3 Department of Infectious Diseases, School of Medicine, Keio University, Tokyo, Japan; Auburn University, United States of America

## Abstract

*Giardia lamblia* is a protozoan parasite that is found worldwide and has both medical and veterinary importance. We applied the transcription start sequence (TSS-seq) and RNA sequence (RNA-seq) techniques to study the transcriptome of the assemblage A WB strain trophozoite. We identified 8000 transcription regions (TR) with significant transcription. Of these regions, 1881 TRs were more than 500 nucleotides upstream of an annotated ORF. Combining both techniques helped us to identify 24 ORFs that should be re-annotated and 60 new ORFs. From the 8000 TRs, we were able to identify an AT-rich consensus that includes the transcription initiation site. It is possible that transcription that was previously thought to be bidirectional is actually unidirectional.

## Introduction

*Giardia lamblia* (also called *Giardia intestinalis*), a member of the family *Hexamitidae,* is a diplomonad parasitic protozoan that infects humans and that was discovered by Leeuwenhoek (1681). *Giardia* has a worldwide distribution and infects a wide range of animals in addition to humans. It is a common cause of diarrhea in both developed and developing countries. For example, *Giardia* is the most commonly detected human intestinal parasite in the United States [1]–[3].

The life cycle of *Giardia* is simple, containing only the trophozoite and cyst stages. The trophozoites are pear shaped, measuring 12–15 µm in length and 4–9 µm in width, with a ventral sucking disk, four pairs of flagella and two identical nuclei. The cysts are oval in shape, measuring 5×7–10 µm, and they contain four nuclei [1], [4]. The cysts are environmentally stable and can survive for weeks to months in water. The cyst is the naturally occurring infective stage [5]. Once reaching the duodenum, the cysts begin to excyst and give rise to trophozoites that attach themselves to the mucosa of the upper part of the small intestine. The symptoms vary widely from no symptoms to acute diarrhea. Although the exact stimulus for encystation remains unknown, trophozoites begin to encyst in the lower part of the small intestine, and cysts passed in stools are already mature and infective [6], [7].

Recurrent infection with *Giardia* occurs when the parasite escapes the immune response of the host due to the expression of variant surface proteins (VSP). *Giardia* has approximately 228 VSP genes, and all of them seem to be transcribed; however, only one is expressed at the cell membrane. The mechanism of selective expression seems to be related to RNA interference and post-transcription regulation. Switching between VSPs leads to changes in the cell wall antigens, allowing the parasite to escape the host’s immunity [8], [9].

There are seven different genetic assemblages known for *Giardia* [10]. Only assemblages A and B infect humans [1]. The genome of *Giardia lamblia* assemblage A was sequenced and published. The assemblage A genome is ∼12 Mb distributed over 5 chromosomes with nearly ∼ 77% representing open reading frames (ORF) with multiple repeats, short intergenic regions, few introns and short untranslated 5′ and 3′ regions (UTRs) [11]. The total count of genes is 9747, and 3766 of these are deprecated genes [12]. *Giardia* has the ability to translate mRNAs with very short 5′-UTRs, which may be as short as one nucleotide (nt) [13]. Most of the 5′-UTRs are less than 20 nt [14]. However, Knodler et al. [15] reported two long 5′-UTRs of 146 nt and 280 nt in an investigation of glucosamine-6-phosphate isomerase expression. The promoter system is thought to be a simple TATA box-like system. A study of a few selected genes suggested that several different sequences could be acting as promoters [14], [16]. Bidirectional transcription is believed to be an inherent feature of *Giardia* [17], leading to an abundance of sterile (non coding) antisense transcripts, which represent approximately 20% of the transcriptome [18]. New evidence suggests that *Giardia* might have some split genes [19], [20].

The availability of second-generation sequencers has made it easier and less expensive to study the gene expression profile of *Giardia*, improving our understanding of the unique features of these parasites and allowing us to identify expressed genes and verify annotated ones. Combining the oligo-capping method [21] and massive parallel sequencing technology, we established a method to collect genome-wide data for transcriptional start sites (TSS). This method is also effective to observe gene expression in a quantitative manner [22].

Another method is RNA sequencing (RNA-seq), whereby cDNA is directly sequenced, allowing for correct identification and quantitation of gene expression and detection of introns [23]. Because *Giardia* has a small genome and only two stages and it is an important human and animal pathogen with a widespread distribution of infection, it is a good target for such analysis. Determining which genes are transcribed and making these data available to the research community is important to understanding the encystation process and the mechanisms by which *Giardia* escapes immunity. Here, we applied both TSS-seq and RNA-seq methods to *Giardia lamblia* assemblage A trophozoites cultured *in vitro*. We aimed to study the relationship between transcription start sites and annotated genes, to confirm known data about *Giardia*, to identify possible new ORFs and to determine whether bidirectional transcription is due to conserved motifs.

## Materials and Methods

### Parasites and Culture

*Giardia lamblia* trophozoites (WB strain, ATCC 50803) of the same strain that was used to obtain the genome sequence were maintained in modified TYI-S-33 medium [24]. Mass culture was performed in 15 ml Falcon™ tubes, and the parasites were collected at a concentration of ∼1, 1×10^6^/ml by centrifugation for at 1500 rpm for 4 minutes. Five volumes of Trizol (Invitrogen, CA, USA) were added to the parasite pellet, mixed by pipetting and then drop-frozen in liquid nitrogen. A pestle and a mortar were used to grind the Trizol pellets to ensure complete destruction of the cell walls and improve the RNA extraction yield. The RNA was then purified.

### Oligo-capping and High Throughput Sequencing

A sample containing 200 µg of the obtained total RNA was subjected to oligo-capping by treatment of the RNA with bacterial alkaline phosphatase (TaKaRa, Japan) and tobacco acid pyrophosphatase (Ambion, USA), followed by ligation with RNA-oligo (5′-AAUGAUACGGCGACCACCGAGAUCUACACUCUUUCCCUACACGACGCUCUUCCGAUCUGG-3′) using RNA ligase (TaKaRa) [22]. The poly A-containing RNA was selected, and first strand cDNA was synthesised using random hexamer primers (5′-CAAGCAGAAGACGGCATACGANNNNNNC-3′) and SuperScript II (Invitrogen). Gene Amp PCR kits (PerkinElmer) were used with the PCR primers (5′-AATGATACGGCGACCACCGAG-3′) (5′-CAAGCAGAAGACGGCATACGA-3′) under the following reaction conditions: 15 cycles of 94°C for 1 min, 56°C for 1 min and 72°C for 2 min. The PCR fragments were size-fractionated and used for the sequencing reactions with the Illumina GA. In total, 36 cycles of the sequencing reactions were performed according to the manufacturer’s instructions.

### Data Processing

The obtained sequences were mapped onto the *Giardia lamblia* ATCC50803 genomic sequencing (version 1.1 *Giardia*db, http://Giardiadb.org/Giardiadb/) with the sequence alignment program Eland. Unmapped or redundantly mapped reads were removed from the data set. Reads with more than two mismatches were also removed. The reads were sorted into TSS sites, and the TSS sites were then clustered. Due to the compactness of the genome and the small intergenic distance, a size window of 10 nts was used to count the TSS sites. Overlapping transcription windows were merged and designated as transcription regions (TRs). TSS reads were considered to be in different TRs if they were separated by more than 10 bases without any TSS reads in between. The start position of a TR or the position of a TSS site with the highest sequence read number was used to evaluate the correlation between TRs and ORFs. For further details, see the supplementary material and methods, figure S1, figure S2, figure S3, and figureS4.

To calculate the background distribution, the Poisson distribution was used. The background distribution was estimated to be ≈1.3 reads for a 10-nt window. In this study, a TR was considered significant if it contained a TSS site with 5 or more reads.

### RT-PCR

To verify the mapping results for the TSS reads, 20 TRs 100 nt downstream from the start site that were more than 500 nt away from any ORF and 10 TRs of 200 nt that were within 500 nt upstream an ORF were chosen to be amplified using RT-PCR. Primer-BLAST was used to design the specific primers [25]. Briefly, SuperScriptII™ (Invitrogen) was used to synthesise the cDNA, and then TaKaRa Ex Taq™ (TaKaRa) was used to amplify the targeted sequences using specific primers for 30 cycles. The primer list is provided in the table S1.

### RNA-Seq

To verify the TSS reads and confirm their relationships to ORFs, high throughput RNA-seq was performed using TruSeq™ (Illumina) according to the manufacturer’s protocol. Briefly, 1 µg of total RNA was purified and fragmented and then used to synthesise the 1^st^ strand cDNA, which was followed by synthesis of the 2^nd^ strand. The ends were repaired, and the 3′ ends were adenylated. The fragments were ligated to adapters and amplified for 15 cycles.

The RNA-seq tags were mapped to the genome. Due to the presence of multiple repeats and the small genome size, Integrative Genomics Viewer (IGV) [26], [27] was used to visually evaluate the expression and verify the TSS reads. Any area of transcription that was not related to a gene was evaluated using NCBI BLAST® [28].

The metagenomic ORF finding tool Orphelia was used to read multiple ORFs in a 700 base pair model to identify ORFs when no ORFs were found within the 500-nt downstream TR [29], [30].

WebLogo, a web-based application that can be used to draw logos for conserved sequences in relation to their positions, was used to detect and draw a sequence consensus [31], [32].

## Results and Discussion

For the first time, we have applied the TSS-seq technique to analyse the TSSs of trophozoites of the assemblage A strain of *Giardia lamblia*, WB clone, ATCC50803. A total of 6,343,253 34-base sequences was generated and mapped to the same strain as that used for genome version 1.1 (http://Giardiadb.org/common/downloads/release-1.1/Glamblia), allowing a 2-base mismatch. Reads were deposited at DDBJ Sequence Read Archive, accession number DRA001089.We obtained 2,600,245 uniquely mapped reads that were sorted into 404,331 TSS sites (the details of the mapping result and sorting are shown in table S2). These reads were clustered into 63,795 transcription regions (TRs) using a 10-base window, of which 8000 TRs were expressed at levels that were significantly higher than the background (see Materials and Methods).

**Figure 1:**
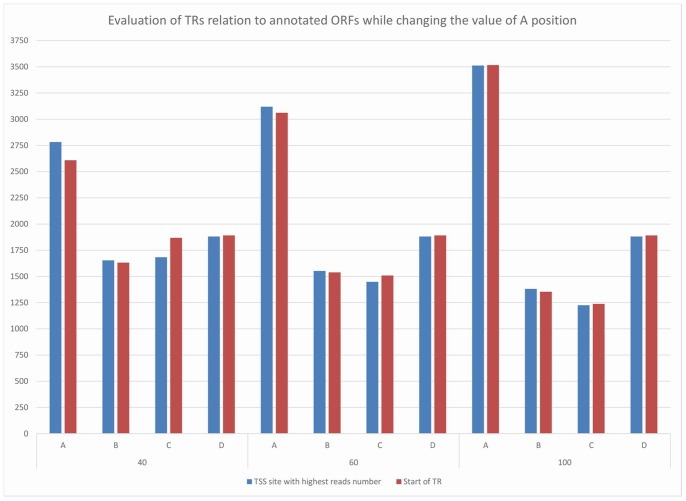
Evaluation of the correlation between TRs and genes. A: Perfectly positioned if the distance between the TR and the start codon is ±40, ±60 or ±100 nt. B: The TR is intragenic. C: If the TR was located between 500 nt up-stream of the first codon and distance A, it was considered as possibly related to the gene. D: If the TR was located more than 500 nt up-stream any annotated ORF.

Next, we evaluated the correlation between the TRs and the ORFs, using the gene file from version 2.1 (http://Giardiadb.org/common/downloads/release-2.1/GintestinalisAssemblageA/), which contains 9747 ORFs. Of these, 3766 ORFs are deprecated. According to the distance from the first ATG, the TRs were categorised into four categories, A, B, C and D (figure 1). In agreement with the previous findings that the majority of the 5′-UTRs are short [14], we found that 2516 (31.5%) TRs out of 8000 are present within 40 nt of the start codons of 2448 of the ORFs. Increasing the distance from 40 nt to 60 nt and 100 nt lead to a gradual increase of 3–5.8% in the number of TRs counted at each step (the details are shown in table S3). We used the position of the TSS site with the highest read count as the reference position of the TRs for this analysis. Using the start position of a TR as a reference position, we obtained a slightly lower number of correlated TRs (2516 TRs to 2448 ORFs using the TSS site with the highest reads compared with 2336 TRs to 2285 ORFs) for TRs located 40 nt upstream. This difference disappeared at a 100-nt distance. Only 1918 (24%) of the TRs were located within ORFs. Of these, 536 were localised within the first 100 nt of the ORF. For the transcripts from these TRs, the translation start site was at the 2nd start codon or later.

**Figure 2:**
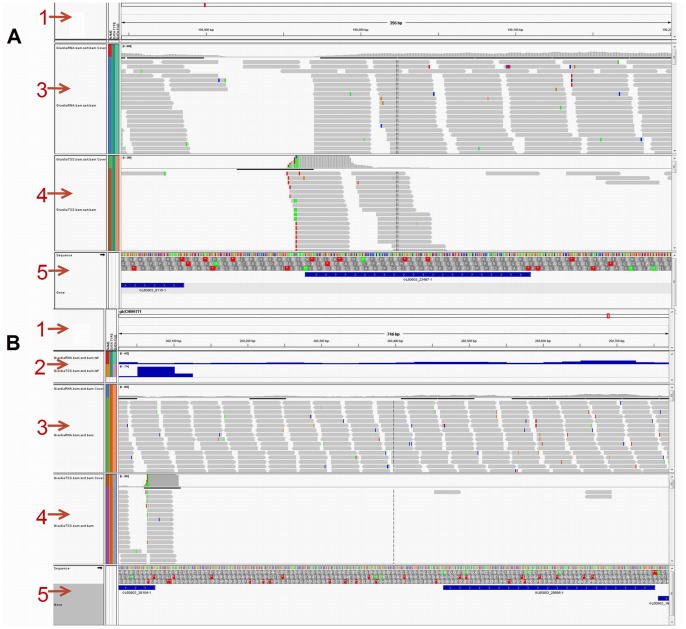
Combining TSS and RNA-seq with the use of IGV tool. A: GL50803_23497 (deprecated gene) has a closely positioned TR and is highly expressed in RNA-seq. B: A long 5`-UTR is observed in GL50803_29595, which is highly expressed in RNA-seq. *Panel formation: 1- Scaffold browser scale. 2- Mapped RNA-seq and TSS-seq read counts in relation to the scaffold. 3- Mapped RNA-seq read distribution. 4- Mapped TSS-seq read distribution. 5- Annotated genes (including deprecated ones).

Interestingly, 3106 (38.8%) TRs were located more than 100 nt upstream of the 1st ATG, and 1881 (23.5%) of these were located more than 500 nt upstream (position D category). The transcripts from these further upstream TRs could correspond to transcripts with long 5′-UTRs or sterile polyadenylated transcripts. Elmendorf et al. [18], estimated that 20% of cDNA libraries are sterile polyadenylated transcripts. These transcripts could have a regulatory importance, either by directly interfering with the transcription of other genes or by initiating the RNAi pathways [8], [9]. Combining TSS analysis with RNA-seq (see below), we were able to identify 7 ORFs with long 5′-UTRs. One of them, GL50803_29595, had a 5′-UTR of 406 bases and was highly expressed in the RNA-seq (figure 2 B). The data are shown in table (1).

**Table 1:** List of genes with long 5′-UTR.

Gene ID	5′-UTR length	Location
GL50803_29096	226-nt long	CH991769∶343,205-343429
GL50803_29595	406-nt long	CH991771∶260,060-260464
GL50803_27713	216-nt long	CH991771∶260,060-260,751
GL50803_28770	151-nt long	CH991768∶89,975-90,125
GL50803_31608	212-nt long	CH991768∶676,797-677,008
GL50803_15887	178-nt long	CH991782∶282,308-282,485
GL50803_32766	178-nt long	CH991814∶234,320-234514

**Figure 3:**
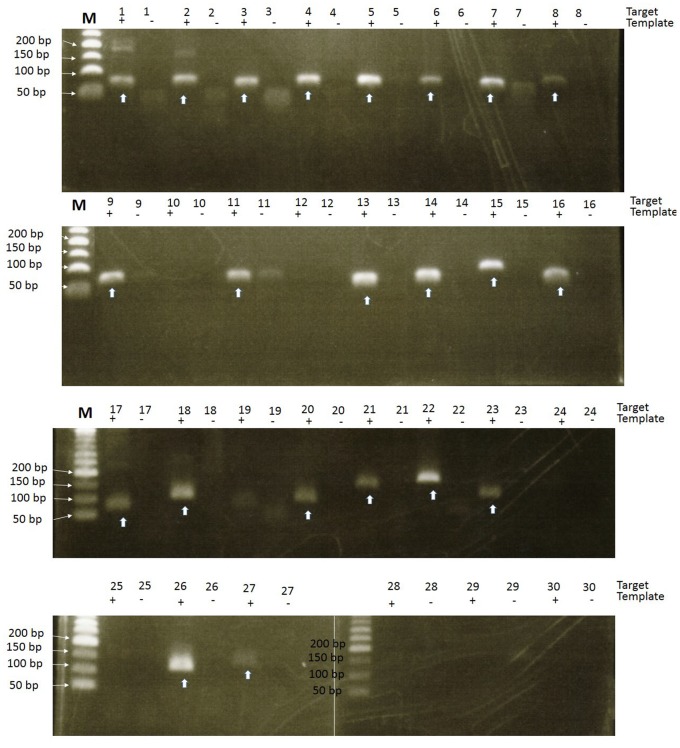
Results of RT-PCR for 30 targets. All samples were run in duplicate (template added and template free). For further details about the position and size of the target, see supplemental material and methods.

We used RT-PCR to verify some of the TSS data. Twenty targets were chosen from TRs located more than 500 nt upstream of annotated ORFs (Position D), and 10 targets were selected from the TRs that were within 500 nt of ORFs (Position A, B or C). In total, 17 out of the 20 position D category TRs and 5 out of 10 of the position A, B or C category TRs were amplified, as shown in figure 3. The finding that 17 targets among the position D category TRs were amplified could be an indicator that some ORFs are present downstream of TRs that are located more than 500 nt upstream of annotated ORFs. It was necessary to investigate whether these tags represent sterile antisense tags or whether they could represent ORFs that were missed during the annotation of genome.

We used Orphelia [29], [30] to check the presence of the ORFs near such TRs. We took 3000 bases of the genome sequence downstream from the start of TRs and examined the presence of ORFs that are 300-nt or more in length. At the same time, we decided to conduct a full-scale RNA-seq to determine whether such ORFs can be expressed or not. To evaluate the RNA-seq data, we first looked at 30 TRs for which we performed RT-PCR. We were able to detect the presence of some transcription for 29 of these 30 TRs. Although we must use caution when interpreting RNA-seq data because they do not have strand specificity (TSS-seq data are strand-specific), RNA-seq can sometimes be more sensitive than RT-PCR. Of the 20 position D category TRs, 19 appeared to have new downstream ORFs that are 100 nt long or more (RNA-seq supports the presence of these transcripts). Two of the TRs had conserved domains and are described below (as re-annotated genes).

The *Giardia* genome contains 9747 ORFs, of which 3766 ORFs are deprecated. We found 4187 ORFs with TRs located from 500 nt upstream of the start codon to the end of ORF. Among them, 554 are deprecated ORFs. A total of 111 of the 575 deprecated ORFs had TRs within 100 nt upstream of the start codon. Thus, these 554 ORFs may be re-considered, or only the 369 ORFs that do not completely overlap with non-deprecated genes. From the RNA-seq data, we could evaluate the expression of 363 of the 575 deprecated ORFs. An example is the deprecated gene GL50803_23497 shown in figure 2 A.

We have identified a total of 84 ORFs that are either novel or that need to be re-annotated by combining TSS and RNA-seq data; omitting ORFs that had no or low similarity or no or low expression. We have examined all of these ORFs using both protein BLAST and nucleotide BLAST. A total of 24 ORFs should be re-annotated. These ORFs have TRs near the proposed start site, and they have RNA-seq tags covering the new annotated regions. Another 60 ORFs are new and were not detected during annotation of the genome. Among these, 13 ORFs belong to the variant surface protein (VSP) family; 5 are re-annotated ORFs and 8 are new ORFs that were expressed according to both TSS and RNA-seq. Another 6 ORFs with Ankyrin-like conserved domains were also found. Using BLAST®, we identified similar genes in either the same 50803 strain or in other *Giardia* strains that have been sequenced (Table 2).

**Table 2:** List of new genes and genes to be re-annotated.

Position	Blast result	Conclusion
CH991763∶266007-267494_R	P23-like domain, similar to GL50581_3538 figGiardiaintestinalis ATCC 50581]	new gene, similar to other assemblage
CH991763∶264707-265966_R	IFT complex B, GL p15, re-annotate GL50803_40995	gene to be re-annotated
CH991817∶18,017-18,463	Hypothetical protein, re-annotate GL50803_25713	gene to be re-annotated
CH991814∶252,697-256,896	Hypothetical protein GL P15/kinase protein	new gene/conserved domain
CH991803∶2702-4543	VSP, similar to GL50803_114065	gene repeat/conserved domain, FU-like and VSP domains,re-annotate GL50803_102540
CH991798∶30,210-30,809	ribosomal protein S11	re-annotate (GL50803_14827) 199AA instead of 154, similarto other assemblage
CH991782∶26,231-28,138_R	VSP, conserved domain	635 instead of 195 AA, original gene GL50803-101380,gene to be re-annotated
CH991779∶250938-252914 -R	VSP, conserved domain	gene repeat
CH991779∶569,674-571,152	VSP, conserved domain	new gene
CH991779∶1,223,042-1,223,698	Hypothetical protein, GL P15	new gene, similar to other assemblage
CH991779∶1,425,747-1,427,552	VSP, conserved domain	gene to be re-annotated, re-annotate GL50803_40630
CH991785∶11,532-11,867_R	SORL conserved domain, hypothetical protein GL P15	new gene
CH991776∶59,721-59,930	ribosomal S30 conserved domain	new gene
CH991771∶171,536-171,874_R(112AA)	similar to hypothetical protein GL50803_32738	gene repeat
CH991769∶78,752-81,292	hypothetical protein, two conserved domains	new gene, similar to other assemblage GL P15
CH991769∶412,442-413,248	similar to reverse transcriptase	gene repeat
CH991769∶624,472-624,627	50S ribosomal protein L39e	new gene
CH991769∶770,102-771,508	hypothetical protein	similar gene/gene repeat, similar to other assemblageGL P15/hypothetical GL50803_17273
CH991768∶744,494-745,936	Hypothetical protein	new gene similar to similar to other assemblageGL P15
CH991768∶1,281,692-1,282,111-R	GL P15, Ribosomal protein S19e domain conserved	new gene-reverse strand, CH991768∶1,281,692-1,282,111-R
CH991764∶147,549-148,025_R	partially similar to hypothetical protein GL50803_20672	new gene
CH991764∶148,024-149,931	VSP conserved domain,	new gene
,CH991762∶115,657-116,463	ANK conserved domain	new gene/gene repeat, similar to GL P15, Ser/Thrprotein kinase
CH991761∶101,085-102,872	VSP conserved domain, similar GL50803_116477	gene repeat, re-annotate GL50803_135831
CH991761∶113,307-113,858	VSP conserved domain	gene repeat
CH991761∶113,432-113,770_R	similar to hypothetical protein GL50803_105806	gene repeat
CH991761∶295,809-301,103	Hypothetical protein, GL P15, conserved domain WD40	new gene, partially similar to Hypothetical protein(GL50803_113673)
CH991763∶4,689-7,532_R	conserved domain, Ankyrin-like and protein kinase	gene repeat, similar to GL50803_113094
CH991763∶689121-689569	partially similar to GL50803_101496	partial gene repeat
CH991763∶688,749-688,942_R	partially similar to GL50803_137676, kinase	partial gene repeat
CH991767∶1667698-1667877	conserved domain, Ferredoxin Fd1, Fd2	partial gene repeat
CH991761∶301,967-303,142	conserved domain, NEK, kinase-like	new gene/gene repeat
CH991761∶302,965-305,298	ANK conserved domain, similar to kinase	new gene
CH991763∶1395469-1397541_R	VSP conserved domain, similar to High cysteine membraneprotein Group 1 (GL50803_91707)	new gene
CH991767∶885323-886138	VSP domain, similar to P15	partial gene repeat
CH991767∶1127974-1130037_R	VSP domain, similar to P15	new gene
CH991767∶1130397-1135277	hypothetical protein, similar to P15	new gene
CH991767∶1135382-1140265	conserved domains, chromosome segregation protein SMC, similar to Axoneme-associated protein GASP-180	gene repeat
CH991767∶1140362-1145860	re-annotate GL50803_32999 to be similar to P15 (GLP15_1881)	many conserved domains
CH991767∶1146036-1147784	conserved ANK domain, Coiled-coil protein [Giardia intestinalis ATCC 50581], Hypothetical protein GL50803_41212	new gene
CH991767∶1696020-1696778	conserved ANK domain and zinc finger, similar to GL50803_113284 hypothetical protein and Protein 21.1 P15	gene repeat
CH991793∶23497-23754	ORF with low similarity	new gene, well expressed in RNA-seq
CH991763∶1,306,665-1,307,180	ORF with low similarity	new gene, expressed in RNA-seq
CH991776∶157233-158693	conserved ANK and kinase domains, partially similar to?NEK (GL50803_93221)	new gene/gene repeat
CH991762∶387,382-387,645	partially similar to hypothetical protein GL50803_38965	partial gene repeat
CH991763∶692,405-692,956	partially similar to GL50803_31921,	new gene
CH991763∶692571-693002	partially similar to hypothetical protein GL50803_5692	new gene
CH991767∶340089-340346	mostly retrotransposon	gene repeat/new gene
CH991767∶436,937-437,701_R	similar to VSP,(GL50803_111732)	gene repeat
CH991767∶435261-437231	similar to high cysteine membrane protein EGF-like (GL50803_114626)	gene repeat
CH991782∶818,861-820,612	similar to P15 and 50581 strains	re-annotate GL50803_40224
CH991761∶20575-22203	similar to P15 and 50581 strains	re-annotate GL50803_96616
CH991767∶1,732,248-1,734,773	similar to P15 and 50581 strains	re-annotate GL50803_39210
CH991779∶262026-264188	similar to P15 and 50581 strains	re-annotate GL50803_35276
CH991776∶21991-23994	similar to P15 and 50581 strains	re-annotate GL50803_34684
CH991779∶1223042-1223698	new hypothetical protein, conserved among 3 assemblages	new gene, expressed in in RNA-seq
CH991769∶937870-939393	new hypothetical protein, conserved among 3 assemblages, conserved Ribophorin I domain	new gene, expressed in in RNA-seq
CH991814∶199061-199591	similar to GL50803_114246, GTP-binding protein, putative	partial gene repeat
CH991779∶1,155,366-1,156,079_R	similar to Rossmann-fold protein [*Giardia lamblia* P15],conserved putative domain	new gene
CH991769∶2,224-3,885_R	similar to hypothetical protein GLP15_2551	new gene
CH991769∶953,848-955,386_R	similar to P15 hypothetical protein	re-annotate GL50803_7035
CH991763∶1385753-1387552_R	similar to hypothetical protein in P15 and 50581 strains	new gene
CH991779∶681,867-683,660_R	similar to P15 and 50581 strains	re-annotate GL50803_2822
CH991776∶278,076-279,610_R	PTZ00382 conserved domain	re-annotate GL50803_97233, well expressed in RNA-seq
CH991769∶77,021-78,700	new hypothetical protein, similar to P15 and 50581	new gene
CH991769∶56,305-56,718_R	new hypothetical protein, similar to P15 and 50581	new gene
CH991767∶707,305-707,724_R	new hypothetical protein, similar to hypothetical protein GLP15_3559	new gene
CH991769∶334626-334943_R	Gene repeat, *Giardia lamblia* ATCC 50803 Pam18p (GL50803_300001)	gene repeat
CH991767∶707,305.707,724_R	similar to hypothetical protein GLP15_3559	new gene
CH991776∶310,552-313,605_R	new hypothetical protein, similar to P15 and 50581	new gene
CH991814∶296,825-303,154_R	new hypothetical protein, similar to P15 and 50581	new gene
CH991769∶494114-494923	new hypothetical protein, similar to P15 and 50581	new gene
CH991814∶275,394-281,945	similar to Kinase [*Giardia lamblia* P15], multiple conserved domain	new gene
CH991779∶986627-987556_R	similar to Kinase GL50803_101307 and GL50803_86934	gene repeat
CH991793∶39014-40039_R	new hypothetical protein, similar to P15 and 50581	new gene
CH991763∶195,962-199,271_R	similar to P15 and 50581 strains	re-annotate GL50803_32861
CH991780∶48,596-52,006_R	similar to P15 and 50581 strains	re-annotate GL50803_41369
CH991763∶487,379-487,747	new hypothetical protein, similar to P15 and 50581	new gene
CH991767∶1652396-1654378	similar to P15 and 50581 strains	re-annotate GL50803_41311
CH991771∶3728-4342	similar to GLP15_4099	re-annotate GL50803_40244
CH991767∶473391-475715_R	similar toGLP15_5080 and GL50581_209	re-annotate GL50803_36426
CH991769∶546414-550226_R	similar to GLP15_5033 and GL50581_4447	re-annotate GL50803_103205
CH991776∶41095-43620_R	similar to GLP15_3901	re-annotate GL50803_39904
CH991768∶596,759-597,880_R	similar to P15 and 50581 strains	re-annotate GL50803_30448

Teodorovic et al. [17] estimated that 50% of transcription loci had bidirectional activity with no correlation between the sense and anti-sense copy numbers. The high frequency of the expected bidirectional transcription suggests that a certain definite bidirectional promoter element is present.

We evaluated our results to detect the bidirectional relationships of the 8000 significantly expressed TRs to the entire set of 63795 TRs. With up to 300 nt difference, we found total of 3686 bidirectional pairs of TRs. Of those pairs, 1175 pairs (2350 TRs, 29.4%) had significant expression in both directions, while 2521 pairs had significant expression in one direction and insignificant expression to the opposite direction. We attempted to determine whether there is any conserved sequence (consensus) for the highly expressed bidirectional TRs by examining the sequences 150 nt upstream and 150 nt downstream from the nucleotide positioned midway between the start sites of each pair. However, we were not able to find any conserved sequences for a promoter region.

**Figure 4:**
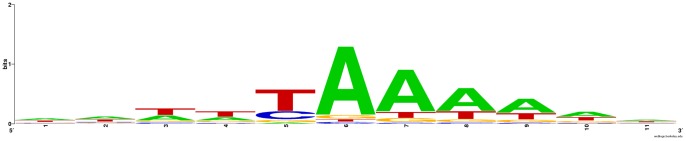
Conserved consensus for the transcription initiation site in *Giardia.*

As the 5`-UTR is very short, this result raised the possibility that there is no real bidirectional transcriptional consensus, but that unidirectional transcription events occur near each other. Thus, we decided to examined the presence of a consensus for the entire 8000 TRs. The use of motif search tools was unsuccessful as *Giardia* lack motifs patterns seen in other eukaryotes so we decide to use alignment tools to find any conserved sequences. Using the start position as a land mark, we failed to find any consensus within 100 nt upstream of the start position of the TR. We thought that the transcription start site with highest copy reads might be the most effective point of reference. We examined 50 nt up stream and 50 nt downstream from that position and identified an AT-rich consensus, which is shown in figure 4. This consensus occurs from −5 nt to +5 nt relative to the position with the highest TSS read number. An A in middle of the sequence should be the major transcription initiation site. This is the first such consensus for transcription initiation to be identified in the genome of *Giardia*. This finding demonstrates the importance of precise mapping of TSS.

We created a weight matrix for this consensus and evaluated the TR regions again. We found that only 565 TRs lack this consensus. Table 3 shows the top 15 repeated variants of the consensus in *Giardia*. A variant of this consensus was predicted by Holberton and Marshall [33] while studying the promoters of cytoskeleton genes. They suggested that the transcription initiation site sequence is composed of nine bases (AATTAAAAA) and is associated with two other sequences of (CAATTT) and (CAAAAA,A/T,T/C,AGA,G/T,TC,C/T,GAA) that they detected using two algorithms and a weight matrix created for seven genes. Additionally, a variant of that consensus (ATTTTAAAAT) was among the sequences suggested by Yee et al., [34] who identified this sequence as the major transcription start site for the glutamate dehydrogenase gene. The authors demonstrated that altering the bases or the order of the bases will lead to severe down regulation of expression.

**Table 3:** Frequent transcription-initiation site consensus variants.

ATTTTAAAATG	21
ATTTTAAAAAT	17
AATTTAAAATG	16
AAAATAAAAAT	15
AAAATAAAATG	13
AAATTAAAAAA	13
AAATTAAAAAT	13
AAATTAAAATG	13
AATTTAAAAAT	13
CTTTTAAAAAT	13
AAAATAAAAAG	12
TTTTTAAAATG	12
AATTCAAAAAA	11
ATTTTAAAAAA	11
AATTTAAAAAA	10
ATTTCAAAAAA	10
ATTTTAATTTT	10

Although many researchers have predicted some sequences such as (CAAT) or (AG) as conserved motifs within 40-100 nt upstream of the transcription initiation site, we did not find any other conserved consensus within 150 nt upstream or downstream of the TSSs with highest reads. Furthermore, we examined up to 300 nt upstream of the TSSs with the highest reads for 565 TRs that lack the transcription initiation consensus. We did not detect any conserved consensus for these 565 TRs. We did find this consensus variant among the sequences reported by Teodorovic et al., [17] at the loci that have been suggested to be bidirectional. Those loci may have multiple transcription initiators rather than one bidirectional promoter.

**Figure 5:**
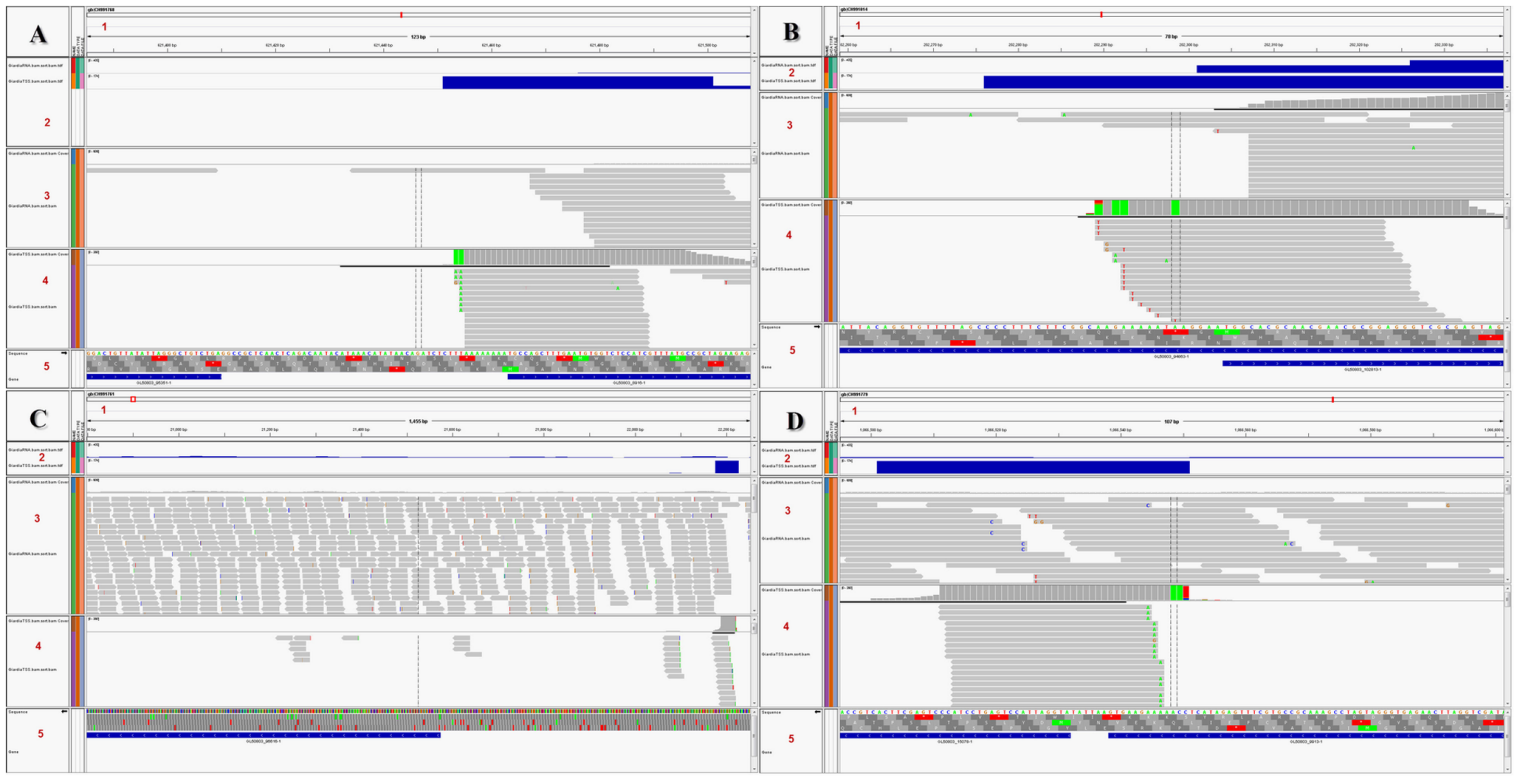
Transcription initiation site with only unidirectional transcription. A: A nearly symmetrical consensus showing only unidirectional transcription. B, C & D: A variant of the consensus showing only unidirectional transcription with the presence of nearby genes. *Panel formation: 1- Scaffold browser scale. 2- Mapped RNA-seq and TSS-seq read counts in relation to the scaffold. 3- Mapped RNA-seq read distribution. 4- Mapped TSS-seq read distribution. 5- Annotated genes (including deprecated ones).

**Figure 6:**
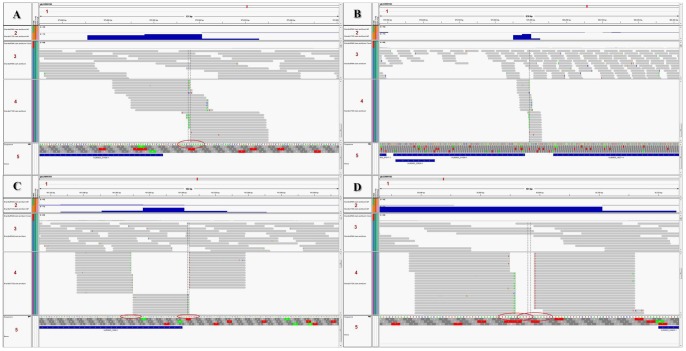
Transcription initiation site with bidirectional transcription. A & B: Bidirectional transcription starting at the same nucleotide position at different distances from nearby genes. C: Bidirectional transcription occurring at the same nucleotide position as one starting at another close transcription initiation site. D: Bidirectional transcription occurring at two overlapping transcription initiation sites. *Red oval mark was used to mark the consensus. **Panel formation: 1- Scaffold browser scale. 2- Mapped RNA-seq and TSS-seq read counts in relation to the scaffold. 3- Mapped RNA-seq read distribution. 4- Mapped TSS-seq read distribution. 5- Annotated genes (including deprecated ones).

As the consensus is somewhat symmetrical, we investigated the possibility of true bidirectional transcription, allowing a ±5-nt difference. We found that 928 pairs of TRs (1856 TRs, 23.2%) were bi-directionally significantly expressed, while the antisense transcripts of 1195 TRs were insignificantly expressed. The occurrence of bidirectional transcription was not related to the symmetry of the consensus, as it is in some cases (figure 5 A); the symmetry of consensus was conserved with no bidirectional transcription. Other variants of the consensus were observed to have only unidirectional transcription (figure 5 B, C & D). In some cases, bidirectional transcription occurred at the same nucleotide within the symmetrical consensus between two adjacent genes (figure 6 A & B). In other cases, bidirectional transcription occurred due to the presence of two nearby transcription initiation sites (figure 6 C) or due to two overlapping transcription initiation sites (figure 6 D).

Combining the TSS-seq and RNA-seq techniques was a powerful approach for identifying new genes, confirming or re-annotating known genes and identifying unusually long 5`-UTRs. TSS-seq allowed us to identify the correct transcription sites, which helped us to find the transcription initiation consensus in *Giardia*. We failed to identify any other motifs in *Giardia*. This raises the question of how transcription starts in other places that lack the transcription initiation consensus. Further work is needed to address this question. The presence of the transcription initiation consensus for the majority of the genes shows how simple yet efficient the transcription mechanism of *Giardia* is.

## Supporting Information

Figure S1 **Mapping of TSS sequence copy and TR clustering.**
(TIF)

Figure S2 **How to measure Distance between Transcription regions (TR) and open reading frames (ORFs).**
(TIF)

Figure S3 **Relation between TR and ORFs if the TR overlap an ORF.** *If ORF1 and ORF2 is in the same orientation: TR3 is Upstream-TR if ATG of ORF1 is nearer than ATG of ORF2. **If ORF1 and ORF2 is in the opposite orientation: TR4 is Always Upstream-TR irrespective nearness of ATG to ORF1 or ORF2
(TIF)

Figure S4 **How to evaluate positions of TRs in relation to ORFs.**
(TIF)

Table S1 **List of targets and primers used in RT-PCR.**
(DOCX)

Table S2 **Statistics of TSS reads.**
(DOCX)

Table S3 **Details of transcription regions (TRs) position in relation to annotated open reading frames(ORFs).**
(DOCX)
