# Supplementary figures and images for: Giardia lamblia Transcriptome Analysis Using TSS-Seq and RNA-Seq
Source: PLoS One. 2013 Oct 7;8(10):e76184. doi: 10.1371/journal.pone.0076184 (PMC3792122; doi:10.1371/journal.pone.0076184)

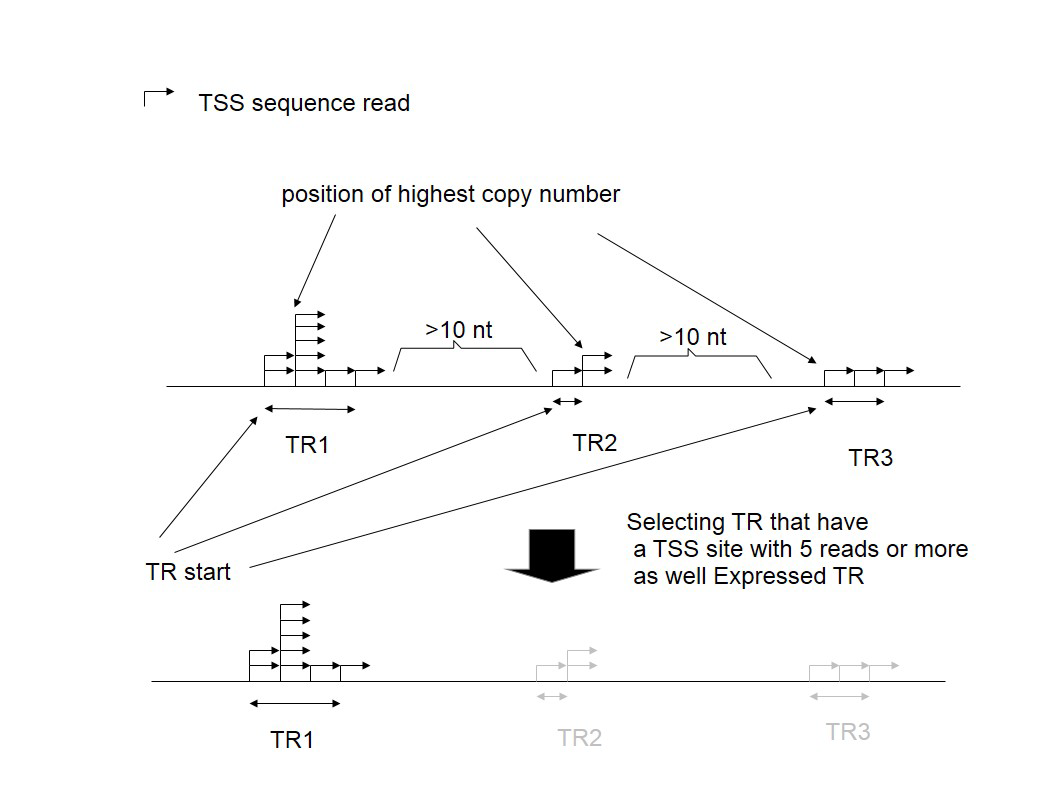

Supplement: Figure S1 — Mapping of TSS sequence copy and TR clustering. (TIF) [file pone.0076184.s001.tif]

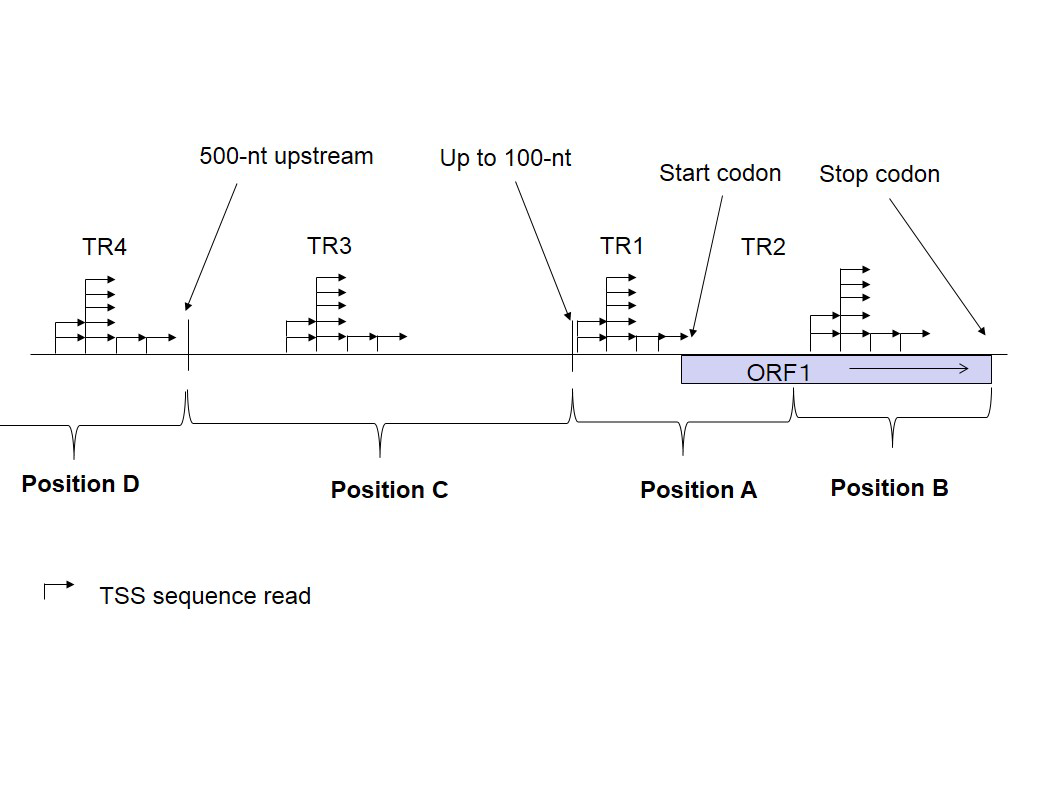

Supplement: Figure S2 — How to measure Distance between Transcription regions (TR) and open reading frames (ORFs). (TIF) [file pone.0076184.s002.tif]

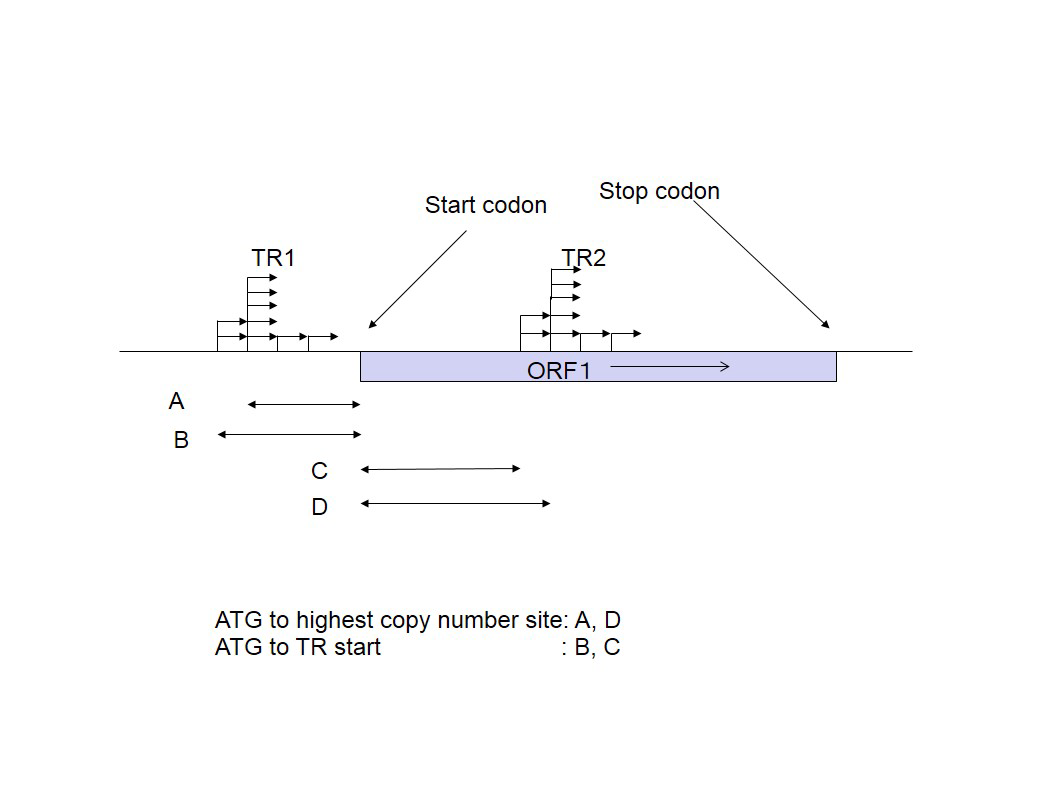

Supplement: Figure S3 — Relation between TR and ORFs if the TR overlap an ORF. *If ORF1 and ORF2 is in the same orientation: TR3 is Upstream-TR if ATG of ORF1 is nearer than ATG of ORF2. **If ORF1 and ORF2 is in the opposite orientation: TR4 is Always Upstream-TR irrespective nearness of ATG to ORF1 or ORF2 (TIF) [file pone.0076184.s003.tif]

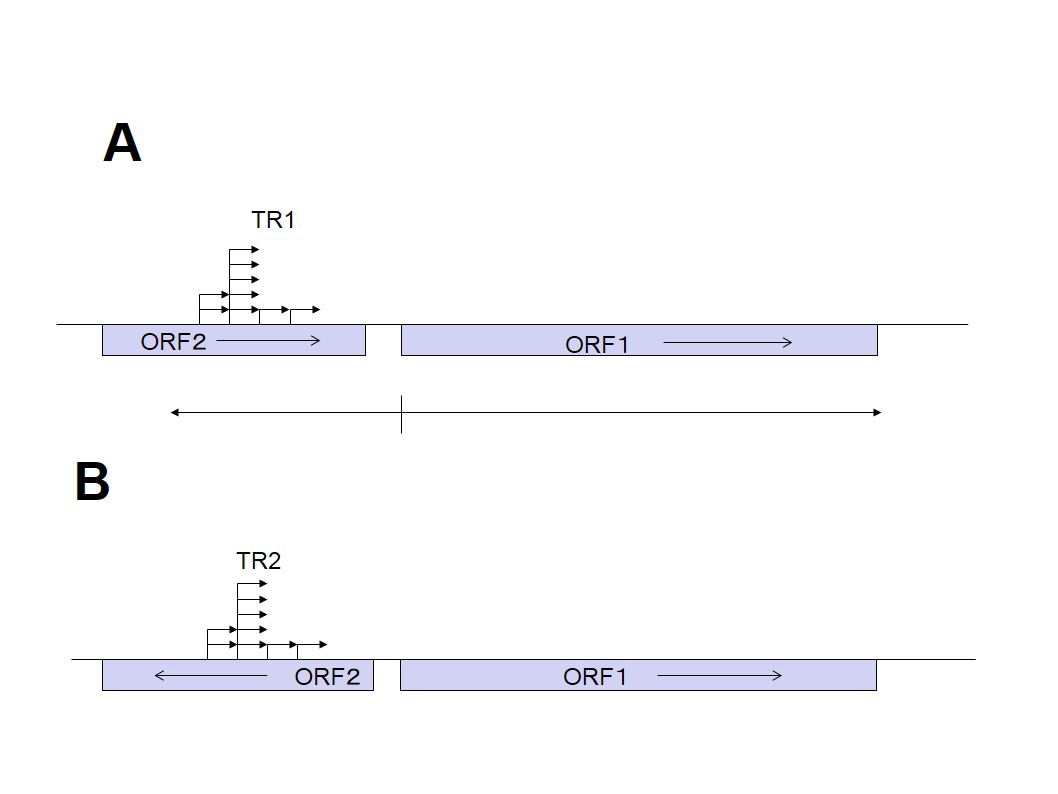

Supplement: Figure S4 — How to evaluate positions of TRs in relation to ORFs. (TIF) [file pone.0076184.s004.tif]
