# Supplementary material for: Giardia lamblia Transcriptome Analysis Using TSS-Seq and RNA-Seq
Source: PLoS One. 2013 Oct 7;8(10):e76184. doi: 10.1371/journal.pone.0076184 (PMC3792122; doi:10.1371/journal.pone.0076184)
Supplement: Table S1 — List of targets and primers used in RT-PCR. (DOCX) [file pone.0076184.s005.docx]

| Target | Position | forward primer Sequence (5'->3') | Reverse primer Sequence (5'->3') | Expected product size |
| --- | --- | --- | --- | --- |
| Category D | | | | |
| 1 | CH991761_43797 to 43897 | AATTATGAGGGCGAAGTGGGCGA | GGCCTTCATGTGCCGCCTCT | 70 |
| 2 | CH991762_145429 to 145529 | AGACAGGAATTAGGAGGGGCGCC | GCGCGCCTCCCTCACGGAAAA | 76 |
| 3 | CH991762_259678 to 259778 | GAACCACAGACCTGTACTGACCCT | GCCTCGGACACGATTGAGTAAAGGA | 72 |
| 4 | CH991763_241985 to 242085 | ATGCACTATCACGTGGAGATGCCC | CGTGCCATGTTTCTACGGGGTC | 77 |
| 5 | CH991767 _ 731939 to 732039 | TATCAGCGCAGCTCTCGCACA | GGGAGTACGGCGACGACTATAAGT | 77 |
| 6 | CH991767_128168 to 128268 | TTCATCGCCCTCCGATCCATTCGT | AGGTAAGACGGGCGGCCGT | 82 |
| 7 | CH991767_1667676 to 1667776 | ATGCCCATCAAGCTCACTGCCTC | ACAGAGACGGGGCACGCGTC | 71 |
| 8 | CH991768_104005 to 104105 | GTTGCTACGAGGCGATGGAGACA | AGGAAGGCTCGGACATCCAAGGA | 87 |
| 9 | CH991768_400323 to 400423 | TGCCGTCACTGTCGATTGTAACCA | ATACGAGCGCGTCGCAGAGG | 86 |
| 10 | CH991768_915269 to 915369 | TCTAAGGCTAACCCAAGTTGTGCCA | TCACAAGCCCCCATGGCTTGC | 72 |
| 11 | CH991769_395238 to 395338 | AGCCTCAACACTTTTCATCAACGCA | AGCTTTGCCTTGGCTGTTAACCC | 84 |
| 12 | CH991769_ 121959 to 122059 | CCAGGGGATCTATTCGCCGGC | AGCGGGTACTTGAGTCTGCAGA | 100 |
| 13 | CH991771-180055 to 180155 | ACTTTGCAGCTCACAGAAAAGGGGT | CACGTTGGGCTCAGCGGTAGC | 70 |
| 14 | CH991779_607940 to 608040 | ATCAGGGTCAAGAAATCTGAAGCGG | AACGCTTCCGTTCGTGATGGTT | 75 |
| 15 | CH991779_1244807 to 1244907 | ATGCAATCCTCTCGCGGCCC | GGCTGCTCATAGGGAAAGGCATGT | 100 |
| 16 | CH991779_1270852 to 1270952 | CAGTCTAGAGTCGCTGGGGGACC | AAGGAGCGGGGTGCAAGCAC | 70 |
| 17 | CH991782_112928 to 113028 | AAGAGCTCAGCTTCCTGGTGGACA | CGATGCGGTGTACGGGCTACAA | 71 |
| 18 | CH991782_368436 to 368536 | CGTCTTCGTTCACAGGTGCTAGCG | GCATGACCGCACGCAGAGTGC | 99 |
| 19 | CH991782_842630 to 842730 | ACAGAGCAGGATAGGGACAGGGACT | TGCCCTCCATCCTAGCCTGGC | 75 |
| 20 | CH991785_11744 to 11844 | AGCCCGACGAGGAGAGGACC | CCGGGTGCATGACCGTAGCG | 99 |
| Category A,B&C | | | | |
| 21 | CH991763_371305 to 371505 | AATGCGCGGCTTTGATGTCTAGC | GGCGCTCGCAGATCTGTGTG | 159 |
| 22 | CH991768_476359 to 476559 | CTGCGATGCGGTGAGGGACG | TGCCAGACCTACAATCCGCCTCA | 188 |
| 23 | CH991779_1065044 to 1065244 | CGCACTAAACCCGGTATCTTCACA | AGCCAGCAGCTCTACACACGT | 125 |
| 24 | CH991782_ 16605 to 16805 | CCACTGGCCTCTCCTGAGGCA | AAGAACGCGAACCCGCCGTC | 166 |
| 25 | CH991782_997416 to 997616 | TTGCCCCGTATGACCCTGCG | CACGCAGTGGGCACCAATGGA | 154 |
| 26 | CH991779_72714 to 72914 | AGCAAGCAGCAAAGTCTTTTGGGC | AACATCCGTCCCACGGCGTC | 119 |
| 27 | CH991769_449991 to 450191 | CGGACGGCGTCTTGCCTTCC | ACAGGACGCACAGGAGCAGTG | 138 |
| 28 | CH991768_1020633 to 1020833 | TGCTACCTTCGGCAACCTCTATGC | GCTCAACGAGACTGTGTGGGCC | 146 |
| 29 | CH991767_ 1789734 to 1789934 | CTCGGGCTCCGCTGAGGACA | ACCTCATCGGACGAGCGCAGA | 134 |
| 30 | CH991763_487340 to 487540 | AGATGGCGACAGAGGTACAGGCT | ACCTCGTCCAGGGCATTCAAGG | 160 |
