# Supplementary material for: Giardia lamblia Transcriptome Analysis Using TSS-Seq and RNA-Seq
Source: PLoS One. 2013 Oct 7;8(10):e76184. doi: 10.1371/journal.pone.0076184 (PMC3792122; doi:10.1371/journal.pone.0076184)
Supplement: Table S2 — Statistics of TSS reads. (DOCX) [file pone.0076184.s006.docx]

|  | TSS sites | TSS reads |
| --- | --- | --- |
| TSS reads that might contain primers | 12,699 | 55,393 |
| TSS reads matching to two positions and had only two base mismatches or less | 27,790 | 144,389 |
| TSS reads matching to more than two positions or had more than two base mismatches | 119290 | 213,496 |
| TSS reads that can be mapped to two positions or more on genome | 142,080 | 357,885 |
| TSS reads uniquely mapped to genome with no base mismatch | 108,657 | 673,555 |
| TSS reads uniquely mapped to genome with one base mismatch | 143,554 | 1,646,178 |
| TSS reads uniquely mapped to genome with two bases mismatches | 152,120 | 280,512 |
| TSS reads uniquely mapped to genome with two mismatches or less | 404,331 | 2,600,245 |
| Total Reads that can be mapped to genome | 559,110 | 3,013,523 |
| Reads that cannot be mapped to genome |  | 3,290,034 |
| Quality control reads |  | 39,696 |
| Total number of 34 bases- long reads |  | 6,343,253 |
