# Supplementary material for: Giardia lamblia Transcriptome Analysis Using TSS-Seq and RNA-Seq
Source: PLoS One. 2013 Oct 7;8(10):e76184. doi: 10.1371/journal.pone.0076184 (PMC3792122; doi:10.1371/journal.pone.0076184)
Supplement: Table S3 — Details of transcription regions (TRs) position in relation to annotated open reading frames(ORFs). (DOCX) [file pone.0076184.s007.docx]

|  | A = 40-nt in relation to TSS site with highest copy reads | Number of related ORF | A = 40-nt in relation to TR start | Number of related ORF | A = 60-nt in relation to TSS site with highest copy reads | Number of related ORF | A = 60-nt in relation to TR start | Number of related ORF | A = 100-nt in relation to TSS site with highest copy reads | Number of related ORF | A = 100-nt in relation to TR start | Number of related ORF |
| --- | --- | --- | --- | --- | --- | --- | --- | --- | --- | --- | --- | --- |
| TRs above threshold at position (A) | 2783 | 2671 | 2608 | 2509 | 3120 | 2931 | 3062 | 2881 | 3512 | 3197 | 3517 | 3189 |
| TRs upstream the 1st ATG | 2516 |  | 2336 |  | 2752 |  | 2697 |  | 2976 |  | 2973 |  |
| TRs downstream the 1st ATG | 267 |  | 272 |  | 368 |  | 365 |  | 536 |  | 544 |  |
| TRs below threshold at position (A) | 3166 |  | 3171 |  | 4433 |  | 4434 |  | 6700 |  | 6704 |  |
| Total TRs at position (A) | 5949 |  | 5779 |  | 7553 |  | 7496 |  | 10212 |  | 10221 |  |
| TRs above threshold at position (B) (located inside the gene) | 1653 | 1323 | 1632 | 1306 | 1551 | 1241 | 1538 | 1236 | 1382 | 1101 | 1354 | 1084 |
| TRs below threshold at position (B) (located inside the gene) | 27782 |  | 27763 |  | 27182 |  | 27172 |  | 26010 |  | 25991 |  |
| Total TRs at position (B) | 29435 |  | 29395 |  | 28733 |  | 28710 |  | 27392 |  | 27345 |  |
| TRs above threshold at position (C) (located within 500-nt upstream till (A) position) | 1683 | 1433 | 1869 | 1587 | 1448 | 1234 | 1509 | 1283 | 1225 | 1062 | 1238 | 1072 |
| TRs below threshold at position (C) (located within 500-nt upstream till (A) position) | 11199 |  | 11198 |  | 10532 |  | 10526 |  | 9437 |  | 9437 |  |
| Total TRs at position (C) | 12882 |  | 13067 |  | 11980 |  | 12035 |  | 10662 |  | 10675 |  |
| TRs above threshold and have same distance as (B) or (C) position |  |  |  |  |  |  |  |  |  |  |  |  |
| TRs below threshold and have same distance as (B) or (C) position | 7 |  | 8 |  | 7 |  | 8 |  | 7 |  | 8 |  |
| TRs above threshold at position (D) (located more than 500-nt upstream any ORF | 1881 |  | 1891 |  | 1881 |  | 1891 |  | 1881 |  | 1891 |  |
| TRs below threshold at position (D) (located more than 500-nt upstream any ORF | 13641 |  | 13655 |  | 13641 |  | 13655 |  | 13641 |  | 13655 |  |
| Total TRs at position (D) | 15522 |  | 15546 |  | 15522 |  | 15546 |  | 15522 |  | 15546 |  |
| Total | 63795 | 4187 | 63795 | 4177 | 63795 | 4187 | 63795 | 4177 | 63795 | 4187 | 63795 | 4177 |
